# Phenylalanine flux and gastric emptying are not affected by replacement of casein with whey protein in the diet of adult cats consuming frequent small meals

**DOI:** 10.1186/s12917-014-0177-8

**Published:** 2014-08-12

**Authors:** Tanya J Tycholis, John P Cant, Vern R Osborne, Anna K Shoveller

**Affiliations:** 1 Department of Animal and Poultry Science, University of Guelph, Guelph N1G 2W1, Ontario, Canada; 2 Procter and Gamble Co., Pet Care, Lewisburg 45338, OH, USA

**Keywords:** Cat, Gastric emptying, Casein, Whey protein, Phenylalanine flux

## Abstract

**Background:**

Decreasing the rate of protein emptying from the stomach may improve efficiency of utilization of dietary amino acids for protein deposition. Some studies in rats and humans have shown casein to be more slowly released from the stomach than whey protein. To test if casein induces a slower rate of gastric emptying in cats than whey protein, L-[1-^13^C]phenylalanine (Phe) was dosed orally into 9 adult cats to estimate gastric emptying and whole-body Phe flux.

**Results:**

Concentrations of indispensable amino acids in plasma were not significantly affected by dietary protein source. First-pass splanchnic extraction of Phe was not different between diets and averaged 50% (SEM = 3.8%). The half-time for gastric emptying averaged 9.9 min with casein and 10.3 min with whey protein, and was not significantly different between diets (SEM = 1.7 min). Phenylalanine fluxes were 45.3 and 46.5 μmol/(min · kg) for casein- and whey-based diets, respectively (SEM = 4.7 μmol/(min · kg)).

**Conclusions:**

In adult cats fed frequent small meals, the replacement of casein with whey protein in the diet does not affect supply or utilization of amino acids. These two milk proteins appear to be equally capable of meeting the dietary amino acid needs of cats.

## Background

Cats are obligate carnivores and require a high level of dietary protein to maintain nitrogen balance, compared to omnivorous and herbivorous species [1]. This high requirement is due to faster rates of catabolism of amino acids. The rate at which dietary protein is emptied from the stomach into the small intestine for absorption may influence the rate of amino acid catabolism and, hence, the ability to meet the cat’s high protein requirement. Casein and whey have been designated slow- and fast-emptying proteins, respectively, in other species. Daniel et al. [2] reported a mean half-time of 78 min for gastric emptying of casein suspensions in rats, compared to 21 min for whey. The slower emptying rate of dietary casein may lead to less accelerated and more prolonged delivery of amino acids to peripheral tissues for deposition of body protein [3]. In contrast, the rapid increase in amino acid absorption from whey resulted in significantly greater oxidative losses of indispensable amino acids in humans [3]. However, Calbet and Holst [4] found no differences in gastric emptying of casein vs. whey suspensions in humans, suggesting that the slow vs. fast designation is not consistent. These differences may be a result of the total intake of the proteins, frequency that proteins and accompanying macronutrients are fed, the form in which they are included in the diet, or the processing methods that these proteins are exposed to. Furthermore, oxidative losses of indispensable amino acids may have less of an effect on protein deposition in cats than omnivores because cats typically catabolize a large proportion of their dietary amino acid intake regardless of frequency of feeding [5].

To our knowledge, the effects of slow vs. fast proteins have not been investigated in cats. To test if casein induces a slower rate of gastric emptying in cats than whey protein, and thus could be an appealing addition to commercial feline diets, we used orally administered L-[1-^13^C] phenylalanine (Phe) to estimate emptying rates and whole-body Phe kinetics in adult cats fed casein- and whey-based diets.

## Methods

### Cats and housing

Nine neutered, specific pathogen-free, domestic shorthair cats owned by Procter and Gamble, Inc. (5 male, 4 female) were used in this study. Cats were 9.5 ± 1.2 years old (mean ± SE) and weighed 5.0 ± 0.4 kg. Standard physical evaluation by the attending veterinarian of the overall health of all cats was completed prior to the start of the study, and they were all deemed healthy. Cats were identified by name and microchip, and housed in the Procter and Gamble Pet Care, Pet Health and Nutrition Center, Lewisburg, OH, USA. Prior to the beginning of the study, cats were acclimated to group housing in the cat colony that was a controlled environment where cats only had indoor access. The cats were exposed to natural and artificial light (from 0600 to 1800 h), indoor environmental temperature was maintained at 22°C, and rooms were cleaned daily. Cats were fed once daily in individual cages within their group housing room. Once the cats completed their feed they were put back down in the group housing environment and all cats completed their daily allotment of food within 5 hrs. If any food was left after 5 hrs, it was weighed and true food intake was calculated. All procedures were reviewed and approved by the P&G Pet Care Animal Care and Use Committee and in compliance with USDA and AALAC requirements. Reporting of methodology in this manuscript adheres to the ARRIVE guidelines.

### Experimental design

Gastric emptying was estimated by comparing the kinetics of labelled Phe excursion through plasma from an oral versus an IV dose [6]. The method also yields estimates of first-pass extraction by the splanchnic bed, and whole-body flux of Phe. Phenylalanine is a dietary indispensable amino acid for protein synthesis and is not catabolized to any extent except in the liver where it is converted to the amino acid tyrosine, which may be incorporated into body protein or further catabolized in the liver to produce ATP or glucose [7]. Because of its low and contained catabolism and small pool size, labelled Phe has often been used as a tracer for measurements of protein synthesis and turnover in animals [8]. Steady, fed-state conditions were used to simplify the calculations of Phe flux. Intravenous Phe kinetics were measured first, and then cats were assigned to casein- and whey-based diets in a crossover design for the assessment of oral Phe kinetics.

Prior to estimation of IV Phe kinetics, all cats were fed a standard commercial adult diet (Iams Multi-Cat, P&G Pet Care, Mason, OH) at 60 g/d once daily at 0700 h for 7 days. This level of intake historically resulted in no weight change in any of the cats and was therefore used as the metabolizable energy requirement for weight maintenance of the cats on this study since we wanted the cats to maintain, not lose or gain, weight. On day 8, after an 18-h fast, Surflo catheters (18 ga × 2”; Terumo Medical Corp., Somerset, NJ) were inserted into one cephalic vein under Propofol sedation (Hospira Inc., Lake Forest, IL). The daily food allocation was divided into 24 small meals. After two small meals were fed 15 minutes apart, baseline blood samples were collected from the catheter and then a bolus of 12 mg/kg BW L-[1-^13^C]Phe (99 atom% 1-^13^C) was administered intravenously (IV) through the catheter and flushed with heparinized saline. Thereafter, cats were fed 1/24 of their daily ration of food every ½ hour to maintain a physiological steady state wherein Phe kinetics would not change during its measurement. Blood samples were collected into heparinized tubes approximately 10, 20, 30, 45, 60, 90, 120, 180, 240, 300, 480, 600, and 720 minutes after the IV bolus. Actual sample times were recorded. Samples were immediately centrifuged at 5000 rpm and plasma was frozen at -20°C for later analysis. Hematocrit was evaluated every 6^th^ sample to ensure that packed cell volume did not diminish. No cats were removed due to a decline in hematocrit.

After the IV Phe study, cats were randomly allocated to isonitrogenous and isocaloric casein- or whey-based diets (Table 1) in a crossover design. The two dry extruded feline diets (Table 1) were made on a twin screw extruder (APV MPF-65, Baker Perkins Limited, United Kingdom) using similar and standard conditioning, extrusion, drying and flavor enhancement processing conditions. Both diets were formulated to meet or exceed Association of American Feed Control Officials (Champaign, IL) recommendations and would be considered “complete and balanced” for adult cats. Cats were maintained on these diets for 23 days, and fed 30 g at 0700 and 1500 h daily. On days 21 and 23, 5 and 4 cats, respectively, were subjected to an oral Phe kinetics protocol according to the IV protocol described above, where 12 mg/kg BW L-[1-^13^C]Phe was administered orally by syringe. Cats were subsequently fed the standard diet for 7 d, switched to the opposite experimental diet for 21 and 23 days, and the oral Phe kinetics protocol was repeated. Assignment of cats to sampling on days 21 or 23 remained the same in both periods.

**Table 1:** Ingredient and chemical composition of casein- and whey-based diets (as-fed basis)

***Ingredients (%)***	**Casein**	**Whey**
Yellow corn	37.2	35.2
Casein	20.0	0
Whey	0	21.6
Chicken Fat	9.7	9.1
Corn gluten meal	6.1	6.2
Chicken by-product meal	10.7	10.7
Chicken	5.0	5.0
Beet Pulp	2.4	2.4
Chicken digest	1.4	1.4
Dicalcium phosphate	1.04	1.05
Corn grits	0.95	0.96
Brewer’s rice	0.94	0.96
Egg	0.81	0.82
Brewer’s yeast	0.76	0.77
Sodium bisulphate	0.76	0.77
Potassium chloride	0.64	0.65
Calcium carbonate	0.64	0.65
Sodium chloride	0	0.48
Mineral Premix^1^	0.42	0.42
Choline chloride	0.20	0.21
Fish oil	0.20	0.20
DL-methionine	0.12	0.12
Vitamin Premix^2^	0.09	0.09
*Analyzed nutrient contents (as fed)*		
Metabolizable energy (MJ/kg)^3^	15.5	16.1
Dry Matter	81.3	82.6
Fat	15.4	17.1
Crude Fiber	1.5	1.2
Ash	6.2	6.2
N-free extract	34.5	34.0
Crude Protein	33.7	34.0
Arginine	1.67	1.80
Histidine	0.76	0.62
Isoleucine	1.39	1.50
Leucine	3.34	3.41
Lysine	2.02	2.07
Methionine	0.97	1.00
Phenylalanine	1.54	1.15
Tryptophan	0.46	0.58
Tyrosine	1.33	0.95
Valine	1.74	1.72

^1^Mineral Premix contained: 40.4% Potassium, 38.1% Chloride, 3 500 ppm Copper, 16 120 ppm Manganese, 60 000 Zinc, 420 ppm Iodine, 150 ppm Cobalt.

^2^Vitamin Premix contained: 36 300 K IU/kg Vitamin A, 1 725 000 IU/kg Vitamin D_3_, 148 650 IU/kg Vitamin E, 22 575 ppm Thiamine, 89 130 ppm Niacin, 19 200 ppm Pyridoxine, 25 935 ppm Pantothenic acid, 2430 ppm Folic acid, 189 ppm Vitamin B_12_, 5520 ppm Inositol, 54 000 ppm Vitamin C, 540 ppm Biotin, 5940 ppm Riboflavin.

^3^Calculated by using modified Atwater coefficients (1).

### Analytical procedures

Concentrations of [^13^C]Phe in plasma were determined with a triple quadrupole mass spectrometer (API 4000; Applied Biosystems/MDS SCIEX, Concord, ON, Canada) coupled to an Agilent 1100 HPLC system (Agilent, Mississauga, ON, Canada; LC-MSMS), as previously described by Turner et al. [9]. For determination of amino acid concentrations, 25 μl plasma were mixed with 200 μl methanol in microcentrifuge tubes. These were spun at 13,000 rpm for 5 minutes. The supernatant was dried under a stream of N_2_, reconstituted in 5 μl 0.1% formic acid in double distilled water and 0.1% formic acid in acetonitrile, and subjected to derivatization with phenylisothiocyanate and separation by HPLC [10],[11].

Nutrient content of the diets were determined on duplicate samples using the AOAC [12] procedures for dry matter (934.01), crude protein (990.03), amino acids (999.12), acid hydrolyzed fat (954.02), crude fiber (969.33) and ash (942.05). The concentration of nitrogen free extract (NFE) was calculated by difference (NFE = 100 – (crude ash + crude protein + acid hydrolyzed fat + crude fiber).

### Estimation of isotope kinetics

Parameters of Phe kinetics and gastric emptying were estimated using methods previously described for dogs [6]. To determine the number of compartments required to simulate Phe elimination from plasma, single $\left(P_{1}e^{-k_{1}t}\right)$ and dual $\left(P_{1}e^{-k_{1}t}+P_{2}e^{-k_{2}t}\right)$ exponential equations were fitted to plasma [^13^C]Phe concentrations following IV dosing (P_V(t)_) using the Solver function of Microsoft® Office Excel® 2007 to minimize residual sums of squares. Curve fits were evaluated based on the root mean square prediction error (rMSPE) as a percentage of the mean P_V(t)_, calculated as:

(1) rMSPE%=∑i=1npredi−obsi2n∑i=1nobsin,

where pred_i_ is the i-th prediction, obs_i_ is the i-th observation, and n is the number of observations. Because the two equations contained different numbers of parameters (q), the decision of which equation best fit the data was based on Akaike’s information criterion (AIC), calculated as:

(2) $$\mathrm{AIC}=\mathrm{nln}\;\left(\sum_{i=1}^{n}{\left(\mathrm{pre}d_{i}-\mathrm{ob}s_{i}\right)}^{2}\right)2q.$$

Phe distribution volume (vol) was calculated from fitted parameter values as:

(3) vol=IVdoseP1+P2.

**Figure 1:**
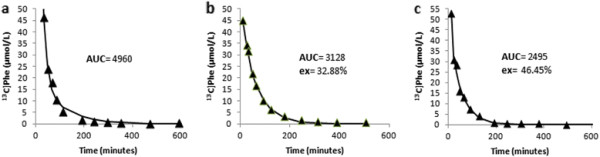
**[**^**13**^**C]phenylalanine (Phe) dilution plots.** Plasma [^13^C]Phe was measured (▲) following **(a)** intravenous or **(b, c)** oral administration of a bolus dose of L-[1-^13^C]Phe into a cat fed either a **(b)** casein- or **(c)** whey protein-based diet. [13C] Phe concentrations were predicted (solid line) with a compartmental model.

**Figure 2:**
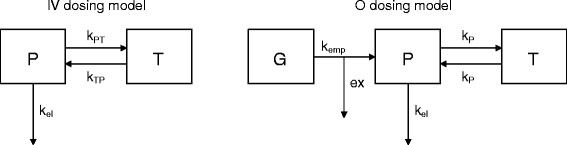
**Compartmental models of [**^**13**^**C] phenylalanine distribution following intravenous (IV) or oral (O) dosing.** Boxes represent state variables, arrows represent flows, P represents plasma, T represents tissue, G represents gut, k_P_ is the rate constant for reversible exchange between plasma and tissue pools, k_emp_ is the rate constant for gastric emptying, ex is the first-pass splanchnic extraction, and k_el_ is the rate constant for irreversible elimination from circulation.

Similar to our previous finding in dogs [6], the analysis identified a two-compartment model as the best fit. Therefore, plasma Phe was assumed to exchange reversibly with a tissue pool (Figure 1). In order to estimate parameters of Phe kinetics from curves of [^13^C]Phe concentrations following oral dosing (P_O(t)_) of the tracer, gastric emptying and first-pass extraction of [^13^C]Phe by the splanchnic bed were considered. The oral dosing model assumes first-order, continuous gastric emptying, 100% post-gastric absorption, and a constant irreversible extraction (ex) of the Phe tracer by the splanchnic bed. Differential equations for the system depicted in Figure 2 are:

(4) $$\frac{\mathrm{dG}}{\mathrm{dt}}=-k_{\mathrm{emp}}G,$$

(5) $$\frac{\mathrm{dP}}{\mathrm{dt}}=k_{\mathrm{emp}}G\left(1-\mathrm{ex}\right)+k_{P}T-k_{P}P-k_{\mathrm{el}}P,$$

and

(6) $$\frac{\mathrm{dT}}{\mathrm{dt}}=k_{P}P-k_{P}T,$$

where G, P and T are [^13^C]Phe concentrations in gut, plasma and tissues, respectively, k_emp_ is the rate constant for gastric emptying, k_P_ is the rate constant for reversible exchange between plasma and tissue, and k_el_ is the rate constant for irreversible elimination from plasma.

The area under [^13^C]Phe concentration curves is related to the dose of [^13^C]Phe injected into the plasma pool and its rate of clearance. Assuming clearance is identical, the ratio of areas under observed P_O(t)_ (AUC_O_) and P_V(t)_ (AUC_V_) curves is equivalent to the ratio of [^13^C]Phe doses introduced into the systemic circulation. Due to the vascular anatomy, entry of orally administered [^13^C]Phe into the systemic circulation requires that it escapes sequestration by gastrointestinal and hepatic tissues of the splanchnic bed, which primarily involves incorporation into secreted proteins and Phe catabolism. Intravenously administered [^13^C]Phe is not subjected to a first-pass splanchnic extraction. Therefore, the value of ex for each cat and diet was estimated from the ratio of AUC_O_ to AUC_V_ as:

(7) ex=1−AUCOAUCV,

where AUC values were estimated using the trapezoidal method.

To estimate k_emp_, k_P_ and k_el_ for each cat and diet, analytical solutions to differential equations 4, 5 and 6 were obtained with Maple 13 software (Waterloo Maple Inc. Waterloo, Canada) and fitted with Excel® Solver to observed P_O(t)_ curves. Gastric emptying half-time was calculated as ln(2)/k_emp_. Whole-body Phe flux was estimated as the product of k_el_, steady-state plasma Phe concentration, and the mean distribution vol estimated from IV Phe kinetics (Eq 3).

### Statistical analyses

Differences between models and diets in goodness of fits and parameter estimates were evaluated by one-way analysis of variance using PROC GLM of SAS (SAS version 9.3; SAS Institute Inc, Cary, NC). Variables that were not normally distributed were natural log-transformed to obtain *P*-values. Values of *P* ≤ 0.05 were considered significant and 0.05 < *P* ≤ 0.10 were considered trends.

## Results

Throughout intravenous and oral Phe bolus protocols, all cats remained healthy, exhibited full food consumption and maintained their body weights. Due to catheter blockage, IV Phe kinetics were not obtained for one cat and results from this animal were not analyzed. Mean plasma indispensable amino acid concentrations in the last 3 samples collected during 1/2-hourly feeding of the diets (Table 2) were not different between casein and whey (*P* > 0.31), although there was a trend for methionine to be lower (*P* = 0.09) and Phe to be higher (*P* = 0.12) with whey. Of the dispensable amino acids in plasma, aspartate and glutamate were higher (*P* < 0.03) on the whey-based diet, while no others were affected (*P* > 0.36).

**Table 2:** **Amino acid concentrations (μ** ***M*) in plasma of adult cats**

**Amino acid**	**Casein**	**Whey**	**Pooled SEM**	***P***
Alanine	654	678	48	0.74
Arginine	133	136	8	0.80
Aspartate	21	38	4	0.01
Citrulline	66	43	8	0.09
Cysteine	28	34	4	0.36
Glutamate	64	88	7	0.03
Histidine	134	122	11	0.45
Isoleucine	90	104	12	0.42
Leucine	174	187	13	0.50
Lysine	218	210	15	0.70
Methionine	139	73	22	0.09
Ornithine	49	56	5	0.38
Phenylalanine	87	103	7	0.12
Taurine	55	57	9	0.87
Tryptophan	103	108	15	0.83
Tyrosine	92	98	14	0.78
Valine	251	301	33	0.31
Indispensable Amino Acids	1242	1123	81	0.37
Dispensable Amino Acids	973	1005	65	0.74
Total Amino Acids	2215	2114	150	0.67

Data are means from 8 to 12 h after initiating consumption of casein- or whey-based diets at 30-min intervals (n = 8).

Modeling the P_V(t)_ curves with a dual exponential equation resulted in lower rMSPE (*P* = 0.04) and AIC (*P* < 0.01) compared with the single exponential equation (Table 3). Lower values indicate better fits. Estimates of Phe distribution volume were not different between equations (*P* = 0.15). A better fit with two exponents indicates two compartments for Phe exchange, which were tentatively identified as plasma and tissue pools. Rate constants for flow from plasma to tissue (k_PT_) and from tissue to plasma (k_TP_) were estimated from the dual exponential fits, according to Shipley and Clark [13], as 0.037 ± 0.008 and 0.041 ± 0.016, respectively. Because these values were not significantly different from each other (*P* = 0.77), it was assumed that a single rate constant (k_P_) could be used to describe the bidirectional exchange between plasma and tissue (Figure 2). Accordingly, P_O(t)_ curves were fit with k_P_ representing plasma-tissue exchange.

**Table 3:** **Fits of 1- and 2-exponent equations to plasma concentrations of [**^**13**^**C]Phe**

	**1-exp**	**2-exp**	**Pooled SEM**	***P***
rMSPE (% of mean)	13.6	2.4	3.6	0.02
AIC	79.4	40.9	5.4	<0.01
Phe distribution volume (L/kg)	0.43	0.31	0.05	0.15

Data are means from 8 curves. rMSPE, root mean square prediction error; AIC, Akaike’s information criterion; Phe, phenylalanine.

Curves of P_O(t)_ were fitted equally well between diets, with no differences in AIC (Table 4). There were no differences between casein- and whey-based diets in k_P_, k_el_, or k_emp_. First-pass splanchnic extraction of Phe was not different between diets and averaged 50%. Peak P_O(t)_ occurred 18.0 and 19.6 min after oral [^13^C]Phe dosing for casein and whey diets, respectively (data not shown). The half-time for gastric emptying averaged 9.9 min with casein and 10.3 min with whey, but diet treatments were not different from each other. Phenylalanine fluxes were 45.3 and 46.5 μmol/(min · kg) for casein- and whey-based diets, respectively.

**Table 4:** **Parameters of Phe kinetics following oral administration of a bolus dose of L-[1-**^**13**^**C]Phe**

	**Oral**		**Pooled SEM**	***P***
	**Casein**	**Whey**		
rMSPE (% of mean)	10.9	12.4	3.0	0.74
AIC	42.4	42.6	7.4	0.91
k_P_ (/min)	0.039	0.063	0.015	0.36
k_el_ (/min)	0.027	0.027	0.003	0.74
k_emp_ (/min)	0.115	0.127	0.053	0.99
first-pass extraction (%)	51.3	48.5	3.8	0.64
gastric emptying half-time (min)	9.9	10.3	1.7	0.87
Phe flux (μmol/(h · kg))	45.3	46.5	4.7	0.85

Data are means from 8 cats per treatment. rMSPE, root mean square prediction error; AIC, Akaike’s information criterion; k_P_, rate constant for reversible exchange between plasma and tissue pools; k_el_, rate constant for irreversible elimination from circulation; k_emp_, gastric emptying constant.

## Discussion

The timecourse of [^13^C]Phe dilution in plasma after IV injection was best described by reversible distribution into two compartments. The volume of distribution was estimated at 31% of body weight, which is larger than the extracellular volume of 20% of body weight estimated by bromide dilution kinetics in cats [14], or 26% of body weight estimated by isothiocyanate dilution in horses [15]. The larger volume of Phe distribution suggests exchange with intracellular fluid and thus, we tentatively ascribe the second Phe compartment to a tissue pool.

According to the ratio of oral to IV AUC, the first-pass splanchnic extraction of Phe in cats was 50% of the oral dose. Splanchnic tissues extracted 45% of an enteral Phe tracer in growing pigs [16]. In adult humans, the first-pass extraction of Phe ranges from 29 to 58% [17]-[19]. However, in adult dogs the first-pass extraction of Phe was approximately 20% [6], which is considerably lower than in cats. Cats are obligate carnivores and their adaptation to high-protein diets may explain why the first-pass extraction of Phe is high [20]. In addition, first-pass extraction of oral amino acids may increase as adults age [21]. In the kinetics study using dogs, the average age was 3 years [6], whereas in our cat study the mean ± SE age was 9.5 ± 1.2 years. The older age of cats may be an additional reason for higher first-pass splanchnic extraction of Phe in cats compared to dogs.

The loss of half of the absorbed Phe as it traverses the splanchnic bed suggests that Phe is supplied in the diet of adult cats at twice the level necessary for protein synthesis in the body. In support of this conclusion, Rogers and Morris [22] noted that half of the Phe supply to kittens could be removed from a diet containing 1.5% Phe with no effect on body weight growth. Total amino acid requirements for body weight maintenance and growth are higher than other mammals because of the chronically high level of basal amino acid catabolism [23]. We did not detect any effect of casein compared to whey protein on the splanchnic extraction of Phe, suggesting that casein did not spare indispensable amino acids from hepatic catabolism as in humans [3].

Estimation of gastric emptying from appearance of an oral [^13^C]Phe dose in plasma is analogous to the acetaminophen absorption test for evaluating liquid-phase gastric emptying [24]. Acetaminophen is poorly absorbed from the stomach and rapidly absorbed in the proximal duodenum so an oral dose appears in plasma according to the rate of delivery from stomach to small intestine [25]. Similarly, Phe is not absorbed from the stomach and absorbed rapidly in the proximal duodenum. Cant et al. [26] derived equations whereby gastric emptying rate constants could be identified from plasma acetaminophen appearance curves, and the same equations were used herein to analyze [^13^C]Phe appearance in plasma.

Labelled Phe reached its maximum concentration in plasma at 19 ± 2 min after oral dosing. Accordingly, the half-time of gastric emptying was estimated to occur at 10 ± 1.7 min, on average. This average is smaller than the mean half-times of 46 to 67 min reported for exit of labelled liquids from the cat stomach [26]-[28] but similar to half-times of 18 to 21 min reported for gastric emptying of suspensions of milk proteins in rats and humans [2],[4]. Our low estimates are likely a consequence of the small meals which are emptied more rapidly from the cat stomach [29],[30]. Gastric emptying measurements are usually performed after a test meal representing 50 to 100% of the daily nutrient intake while we offered only 1/24 of the daily intake with the gastric emptying tracer. The large meal size in gastric emptying studies is used as part of a standardized protocol to allow clinical detection of pathological or pharmacological abnormalities in individuals [24]. Our objective, on the other hand, was to compare amino acid supply from casein- and whey-based diets and, in order to measure amino acid flux, we maintained a non-isotopic steady-state of amino acid concentrations by feeding frequent small meals throughout the day. Although the small meals may have accelerated gastric emptying, there was opportunity to detect differences between diets because, according to our k_emp_ estimate of 0.12/min, 97% of the [^13^C]Phe dose was cleared from the stomach in the 30-min interval between meals. Furthermore, this method of “nibbling” is thought to be normal for cats that are free fed and a benefit to maximize digestibility and avoid insulin resistance [31].

Including casein in the diet of frequently fed adult cats did not slow down liquid-phase gastric emptying, which could account for the lack of effect on splanchnic extraction of Phe. Calbet and Holst [4] also found no difference in liquid-phase gastric emptying between casein and whey protein in humans, while Daniel et al. [2] reported an increase in half-time from 21 to 78 min in rats given casein instead of whey protein. Hall et al [32] found that a liquid casein meal stimulated faster release of acetaminophen from the stomach of humans compared to liquid whey protein. Therefore, the designation of casein as slow and whey protein as fast is not consistent across studies or species.

Phenylalanine flux was not affected by substituting casein for whey protein in the diet. Flux represents entry of Phe into the plasma pool from post-splanchnic absorption plus breakdown of proteins in the body, or exit from the plasma pool to whole-body protein synthesis plus catabolism, which is primarily hepatic. Assuming 90% digestibility and 50% first-pass splanchnic extraction, entry rates of Phe from the diet were 41 and 31 μmol/(h · kg) for casein and whey treatments, respectively, equivalent to 90 and 67%, respectively, of the flux estimates. These proportions are overly high, as intake normally accounts for 25 to 40% of flux in various species [33], and was 50% in adult cats [34]. Phenylalanine flux was estimated from k_el_, Phe concentration in plasma, and Phe distribution volume, so imprecision in any one of these variables could contribute to the underestimation. [^13^C]Phe curves were well fitted with a monoexponential decline yielding k_el_ estimates that exhibited low variability across cats and diets. Thus, k_el_ estimation does not appear suspect, nor do the Phe concentrations in plasma which are similar to previously reported values [35]. The distribution volume estimate of 0.31 L/kg is similar to values of 0.34 and 0.47 L/kg estimated for leucine distribution in humans [36],[37], but is approximately 5 times smaller than the methionine distribution volumes of 1.2 to 1.8 L/kg estimated in rats [38]. If Phe distribution volume were 1.2 L/kg, the flux estimate would be 178 μmol/(h · kg), on average.

## Conclusions

While there is uncertainty about the true Phe distribution volume and Phe flux, it does not affect the conclusion that diet had no effect on Phe flux. This finding is consistent with the lack of effect on liquid-phase gastric emptying, first-pass splanchnic extraction of Phe, and indispensable amino acid concentrations in plasma. In adult cats fed frequent small meals, the replacement of casein with whey protein in the diet does not affect systemic supply or utilization of amino acids. These two milk proteins appear to be equally capable of meeting the amino acid needs of cats. Further research should investigate other purported benefits of casein and whey, such as its effects on carbohydrate metabolism and palatability [39].

## Competing interests

AKS is an employee of The Procter and Gamble Co. that funded the study.

## Authors’ contributions

TJT analyzed the data, conducted the model fits, and drafted the manuscript. JPC helped with study design, modelling and data analysis, and editted the manuscript. VRO participated in data analysis and revision of the manuscript. AKS conceived the study, supervised treatments and sample collection, collated data and editted the manuscript. All authors read and approved the final manuscript.
